# Applying a Multi-Dimensional Digital Food and Nutrition Literacy Model to Inform Research and Policies to Enable Adults in the U.S. Supplemental Nutrition Assistance Program to Make Healthy Purchases in the Online Food Retail Ecosystem

**DOI:** 10.3390/ijerph18168335

**Published:** 2021-08-06

**Authors:** Katherine Consavage Stanley, Paige B. Harrigan, Elena L. Serrano, Vivica I. Kraak

**Affiliations:** 1 Department of Human Nutrition, Foods, and Exercise, Virginia Polytechnic Institute and State University (Virginia Tech), Blacksburg, VA 24061, USA; paigeharrigan@vt.edu (P.B.H.); serrano@vt.edu (E.L.S.); vivica51@vt.edu (V.I.K.); 2 Virginia Family Nutrition Program, Virginia Tech, Blacksburg, VA 24061, USA

**Keywords:** online food retail, food retail environment, online shopping, federal nutrition assistance, SNAP, nutrition literacy, food literacy, digital literacy, policy, COVID-19

## Abstract

The United States (U.S.) Department of Agriculture (USDA)-administered Supplemental Nutrition Assistance Program (SNAP) made substantial changes in response to the coronavirus disease 2019 (COVID-19) pandemic. These changes highlight the need to identify the digital literacy skills and capacities of SNAP adults to purchase healthy groceries online. We conducted a scoping review of four electronic databases, Google and Google Scholar to identify studies that measured food and nutrition literacy outcomes for U.S. adults. We applied a multi-dimensional digital food and nutrition literacy (MDFNL) model to assess six literacy levels and components. Of 18 studies published from 2006–2021, all measured functional and interactive literacy but no study measured communicative, critical, translational, or digital literacy. Six studies examined SNAP or SNAP-Education outcomes. Adults with higher food or nutrition literacy scores had better cognitive, behavioral, food security and health outcomes. We suggest how these findings may inform research, policies, and actions to strengthen the multi-dimensional literacy skills of SNAP participants and SNAP-eligible adults to support healthy purchases in the online food retail ecosystem.

## 1. Introduction

The severe acute respiratory syndrome coronavirus 2 (SARS-CoV-2) that caused the coronavirus disease 2019 (COVID-19) spread throughout the United States (U.S.) and globally in late 2019 through 2021. By June 2021, COVID-19 had infected over 33 million Americans and caused more than 599,000 deaths [1]. During 2020, COVID-19 profoundly weakened the food and nutrition security for American households. One in four urban U.S. households with children experienced food insufficiency and adverse mental health outcomes [2]. Many Americans struggled to pay their monthly rent, credit card bills, or other debts, and depleted their savings accounts or could not afford medical care [3,4]. By December 2020, 11 million Americans had transitioned into poverty after losing their jobs and depleting the short-term emergency funds provided by the federal government through the 2020 Coronavirus Aid, Relief, and Economic Security (CARES) Act [5,6]. By March 2021, 18 million adults reported not having enough food and 67 million adults had difficulty paying for household expenses, such as rent, food or medical costs [7].

COVID-19 disrupted the U.S. food supply, distribution, and services systems and produced major changes in the federal government’s safety-net programs [8]. The Supplemental Nutrition Assistance Program (SNAP), administered by the U.S. Department of Agriculture (USDA), provides monthly monetary benefits to low-income families with a household income of ≤130 percent of the poverty income level. SNAP served 40 million Americans in 2019 and cost U.S. $70 billion [9].

Prior to the COVID-19 pandemic, U.S. household food insecurity rates were about 10.5 percent in 2019, representing 13.7 million households and 35 million Americans. These rates were higher for households with children, with 13.6 percent of households facing food insecurity and millions of children who experienced disrupted eating and hunger [10,11]. These pre-pandemic food insecurity rates were the lowest documented since the early 1990s [12]. By June 2020, food insecurity among low-income U.S. households with children had tripled to nearly 30 percent [13]. The Bipartisan Center for Budget on Policy Priorities estimated that 25 million Americans were unemployed by December 2020, and every U.S. state had experienced an increase in SNAP enrollment, especially among Black and Latino households that reported having inadequate food [7]. Food insecurity is projected to decline in 2021; however, these rates may remain higher than pre-pandemic levels, and about 42 million people may not have adequate food [12].

COVID-19 shifted the way that Americans access, purchase, prepare and eat food. In response to the pandemic and the resulting restrictions and safety precautions, Americans made fewer in-person grocery shopping trips; used online cashless e-commerce platforms to order food and groceries for delivery or pick up; prepared and ate more meals at home with their families; stockpiled a higher proportion of shelf-stable, frozen, and highly processed food products or prepared meals; and were exposed to more direct-to-consumer branded product advertising through online digital marketing platforms [14,15,16,17]. Online shoppers reported purchasing less fresh produce and more unhealthy processed products promoted by retailers through digital technology [18,19] that contribute to poorer diet quality and health. Online food shopping is expected to grow, as marketing research has forecasted that 70 percent of Americans may purchase a proportion of their groceries online by 2024, representing U.S. $100 billion annually in online sales [20].

### 1.1. SNAP Expansion Allows Online Food Grocery Shopping

The Families First Coronavirus Response Act of 2020 provided the USDA Secretary with the authority to approve state agency waivers to change the emergency SNAP eligibility standards and provide pandemic electronic benefits transfer (P-EBT) benefits to eligible Americans [21]. In response to COVID-19, the USDA also expanded the SNAP Online Purchasing Pilot program, funded by the 2014 U.S. Farm Bill in five states (i.e., New York, Washington, Alabama, Iowa, and Oregon). This expansion allowed SNAP participants in 47 states and the District of Columbia to use their benefits for online grocery shopping at approved retailers [22,23]. Amazon and Walmart, for example, are major USDA-authorized online grocery retailers that serve SNAP recipients in most states [23]. Online orders can be picked up curbside (called “Click and Collect”) or delivered to SNAP participants’ homes. However, SNAP funds cannot be used for delivery or other food purchasing fees [24]. President Biden signed the American Rescue Plan into law in March 2021, which included an extension of the 15 percent increase in SNAP maximum benefit allotments through September 2021. The law also provides U.S. $1.15 billion to help states manage SNAP programs and U.S. $25 million to improve SNAP online purchasing, including mobile EBT use and retailers’ technological capacities [25].

Before 2020, online grocery shopping represented less than three percent of SNAP users’ spending [26]. SNAP recipients reported many barriers to online shopping, including a lack of trust in the online retail process, a perceived lack of control over food selection, and higher prices for products purchased online instead of in brick-and-mortar stores. However, convenience, free shipping, and discounts may motivate SNAP participants to use their P-EBT cards to purchase groceries online [26]. Due to the rapid expansion of the SNAP Online Purchasing Pilot Program during the COVID-19 pandemic, there has yet to be a formal evaluation of the program. A USDA Economic Research Service study released in July 2021 found that as the Pilot program expanded, the value of SNAP benefits used for online grocery purchases grew from $2.9 million USD in February 2020 to $196.3 million in September 2020 [27]. More than 1.5 million households were purchasing groceries online with SNAP benefits by March 2021 [28].

### 1.2. Digital Infrastructure and Access and Digital Literacy in the U.S. Population

Affordable access to broadband internet services is a social determinant of health [29], yet an estimated 21.3 to 42 million Americans do not have access to broadband internet services [30]. Americans living in rural areas are less likely than urban or suburban adults to have broadband at home or own a smartphone [31]. In 2019, less than a third of U.S. adults with an annual income below U.S. $30,000 did not own a smartphone and more than 40 percent did not have broadband services, a traditional computer, or were not a tablet owner. About a quarter (26 percent) of Americans living in low-income households are “smartphone-dependent internet users,” which means that they own a smartphone but do not have broadband services at home [32]. Sharing a single smartphone or mobile device involves multi-tasking many activities, such as ordering groceries or paying bills online; it is particularly challenging for adults supervising children who are learning remotely at home due to COVID-related school closures [32].

The digital divide disproportionately affects low-income and racially or ethnically diverse older adults, non-native English-speakers, the disabled, and senior citizens who do not have access to high-speed 4G or 5G broadband internet services or lack the skills to use digital technology. Therefore, daily activities, such as scheduling telemedicine appointments with healthcare providers, buying groceries, or paying bills online, are affected [33,34]. The Pew Research Center reported that Black and Hispanic adults are less likely than White adults to own a computer or have high-speed internet services at home, although smartphones are reducing these disparities [35].

The United Nations Nutrition defines digital literacy as “the ability and skills to find, evaluate, create and communicate information effectively using digital technologies and platforms” [36]. The growth in digital technology is an opportunity to use digital platforms and channels to improve nutrition and health and transform the food system. It also presents new challenges ranging from cybersecurity and data privacy risks to the increased targeted online marketing of unhealthy food and beverage products.

### 1.3. Food and Nutrition Literacy of Adult SNAP and SNAP-Education Participants

Evidence shows that an increase in SNAP benefits may improve the food security status of low-income households, but not necessarily improve overall diet quality [37]. Research also suggests that the dietary intake and diet quality of low-income SNAP participants do not align with the Dietary Guidelines for Americans (DGA) [38,39]. This observation is important because SNAP participants experience higher rates of obesity, type 2 diabetes, and cardiovascular diseases [40] that complicate COVID-19 morbidity, exacerbate food insecurity, and increase mortality rates [41]. Research suggests that low-income Americans who are more confident in their food resource management [42] and their financial literacy skills [43] are less likely to be food insecure. Low numeracy skills are also associated with higher body mass index and lower healthy-weight management [44].

SNAP-Education (SNAP-Ed) is a federal program that supports SNAP participants by providing nutrition education, including food resource management and access to affordable and nutritious foods and beverages [45]. SNAP-Ed’s best-practice guidelines recommend that educational materials should consider literacy, especially age and reading level [46]. However, neither SNAP nor SNAP-Ed provides any clear guidance or recommendations for improving health, digital, food, nutrition, and/or financial literacy among SNAP-eligible adults. The COVID-19 and post-COVID online food shopping and eating trends underscore the need for SNAP participants at risk of food insecurity to develop many literacy skills to navigate the shift from the in-store to online path to purchase food ecosystem in order to make healthy food and beverage product choices that align with national food guidance including the 2020–2025 DGA [47] and USDA’s MyPlate [48].

### 1.4. Study Purpose

**Figure 1:**
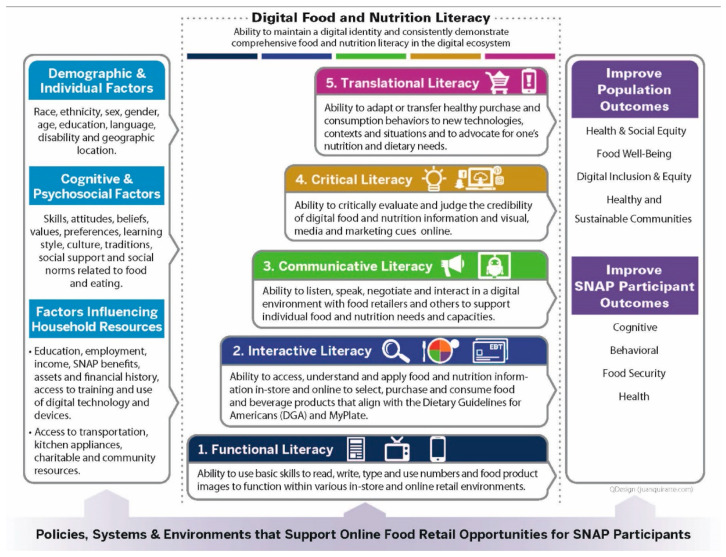
Multi-dimensional Digital Food and Nutrition Literacy (MDFNL) model to support SNAP adults to make healthy purchases in an online food retail ecosystem [49].

The aim of this study is to apply a newly developed Multi-Dimensional Digital Food and Nutrition Literacy (MDFNL) model [49] (Figure 1) to examine the capacities and skills of SNAP participants and SNAP-eligible adults (herein after referred to collectively as SNAP adults) to make healthy food and beverage purchases online. The MDFNL model offers five progressive food and nutrition literacy levels (i.e., functional, interactive, communicative, critical, and translational literacy). It also depicts digital literacy as a cross-cutting factor for all levels. This study assesses how food and nutrition capacities and outcomes have been measured for U.S. adults compared to the MDFNL model. The study aims to identify gaps in the policies, systems, and environments to support the online food retail opportunities for SNAP adults. The findings are used to recommend actions for diverse U.S. actors to strengthen the digital food and nutrition literacy infrastructure and skills for SNAP adults.

## 2. Materials and Methods

This study was guided by two research questions (RQ):

RQ1: What does the available evidence show about the literacy capacities and skills of American adults, especially low-income SNAP adults, to make healthy and affordable purchases within the online food retail ecosystem?

RQ2: How can the MDFNL model be used to assess the available evidence on U.S. adults’ literacy capacities and skills for online purchases to inform future policy and research?

The study used a systematic scoping review process, guided by a restricted review framework [50], to compile and analyze relevant evidence. The five stages of the Arksey & O’Malley 2005 [51] framework for conducting a scoping review was used to identify, select, compile, and analyze evidence that was synthesized into a narrative summary.

The first author, K.C.S., worked with university librarians to develop the search strategy to compile data to inform RQ1. The Preferred Reporting Items for Systematic Review and Meta-Analysis Protocol (PRISMA-P) [52] and the PRISMA Extension for Scoping Reviews (PRISMA-ScR) [53] checklists were used for the search strategy. Risk of bias and study quality were not assessed since the research questions were exploratory in nature. The title and abstract searches were conducted across four electronic databases: Academic Search Complete, PsycINFO, PubMed, and Web of Science. Google (first 200 hits) and Google Scholar (first 200 hits) [54] were also searched in incognito mode to collect data to inform the research questions. The following search terms were used for each of the databases and platforms: (literacy) AND (nutrition* OR food OR diet*). Where applicable, relevant MeSH terms (i.e., literacy; food; diet) were also included in the search. Peer-reviewed and gray literature sources published from journal inception to January 18, 2021, were considered for inclusion. Table 1 summarizes the population, intervention, comparison, outcome, time, and setting/study design (PICOTS) criteria used to identify observational, cross-sectional, or intervention studies that reported relevant food or nutrition literacy outcomes for U.S. adults.

The search identified primary evidence sources that assessed nutrition or food literacy characteristics and cognitive, behavioral, food security, and/or health outcomes relevant to understanding the capacities and skills of SNAP adults operating in the online digital food retail ecosystem. K.C.S. conducted the electronic searches, screened, and identified relevant full-text articles for consideration. Two co-authors, K.C.S. and P.B.H., independently reviewed the full-text articles for alignment with the PICOTS criteria and discussed or resolved any discrepancies related to the interpretation of evidence sources. P.B.H. conducted the data extraction and created an evidence table to summarize the lead author and year published; the study objective; the population and location; the study design and data collection period; the cognitive, behavioral, food security, and/or health outcomes; and the major study findings. The lead author (K.C.S.) developed a separate evidence table to summarize the self-reported type of literacy assessed; the literacy tool used; the literacy capacities and skills measured; and the level of literacy measured based on the MDFNL model [49].

## 3. Results

**Figure 2:**
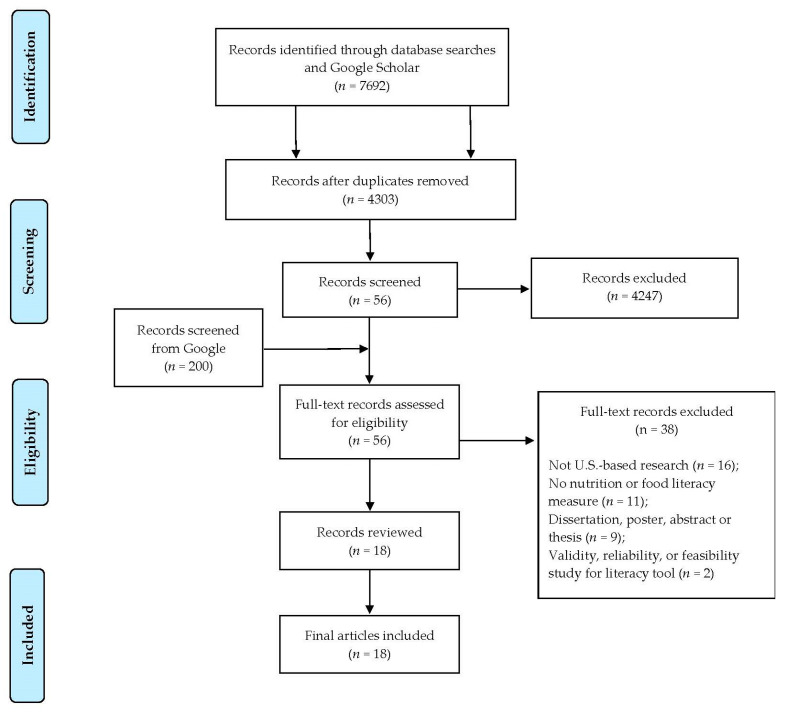
PRISMA flow diagram for the scoping review of studies that evaluated the food and/or nutrition literacy capacities and skills of U.S. adults.

Figure 2 describes the PRISMA flow diagram that identified 18 studies [55,56,57,58,59,60,61,62,63,64,65,66,67,68,69,70,71,72] published between 2006 and 2020. These included 15 observational or cross-sectional studies and 3 intervention studies. Eleven studies were conducted in Florida, Indiana, Kansas, Louisiana, Maryland, New York, Tennessee, and Texas [58,59,60,61,62,63,65,67,68,69,70]; four studies were conducted in the mid-Atlantic [66], Southeast [57], or the lower Mississippi Delta regions [71,72]; and three were national studies [55,56,64], two of which were administered via mail [55,64].

### 3.1. Overview of Studies That Evaluated Food and/or Nutrition Literacy of U.S. Adults

Table 2 outlines the objectives, location, population, and outcomes measured and the major relevant findings for each of the 18 studies. Only three of the 18 studies [55,63,70] described using a conceptual or theoretically grounded framework to inform the study design and interpret the results. Grutzmacher et al., 2020 [59] noted the importance of using conceptual frameworks in future studies to ensure that similar domains are not conflated and to improve measurement. Three studies [56,61,62] assessed the food security status of either individuals or households, making it difficult to generalize the relationship between literacy and food security status. These studies did not consistently use the same eligibility criteria; therefore, it is difficult to compare the results across literacy and income levels. Six studies [56,58,59,62,68,72] reported including low-income SNAP or SNAP-eligible participants. While several studies included diverse racial and ethnic participants, the majority of participants were White. Fifteen of the 18 studies reported results for two or more racial or ethnic groups [55,56,58,60,61,62,63,64,65,66,67,68,69,71,72], and seven of these studies [55,62,64,66,67,68,69,72] found a statistically significant association between race and/or ethnicity and a food and/or nutrition literacy outcome.

No study measured any component of digital literacy or the online digital retail environment more broadly. Therefore, the digital literacy components in the MDFNL model [49] were not included in this assessment. Based on the MDFNL model’s definitions and principles, all studies (*n* = 18) measured functional and interactive literacy. However, no studies assessed more advanced literacy skills as reflected in the communicative, critical, and translational levels of the MDFNL model (Table 3).

Zoellner et al., 2009 [71] was the only study that reported on media used by participants to obtain health, food, and dietary information. Only one study [56] reported measuring financial literacy skills of SNAP participants. Six studies measured one or more household resource management skills, such as frugal buying and stretching food dollars (e.g., making a grocery list and using coupons or other means of savings) [56,58,61,65,69] or extending the food safety of perishable items through refrigeration and being aware of the cost of organic foods [57].

### 3.2. Literacy Measurement and Tools

Several different methodological instruments and tools were used to assess health, food and/or nutrition literacy of the adult populations studied (Table 3). The Newest Vital Sign (NVS) health literacy tool was used in eight of 18 studies [57,59,63,66,68,70,71,72]. The NVS tool was validated as a rapid six-item health literacy screener to be administered by an interviewer and not self-administered online [73]. The NVS tool measures an individual’s understanding and comprehension of a food product label for ice cream. Our review found that three studies used a shortened NVS tool version (four rather than six questions) [55,59,64] and two studies [55,64] used the shortened NSV tool in a mailed survey format. We found that the NVS tool has been used beyond its intended function as a health literacy screening tool to also serve as a proxy measure of food and nutrition literacy. The NVS tool measures a limited type of nutrition literacy that aligns with the functional and interactive literacy levels within the MDFNL model [49,74,75,76].

## 4. Discussion

This study assessed 18 studies that reported on the nutrition and/or food literacy status of American adults and the relevant cognitive, behavioral, food security, and/or health outcomes reported, using categories from the MDFNL model [49]. We used the MDFNL model to describe the levels of literacy assessed by each study based on the participants’ capacities and skills tested. This study also identified knowledge gaps to support U.S. adults’ multi-dimensional literacy skills. Finally, it examined the context needed to support digital food and nutrition literacy proficiency and improve both individual and community outcomes. The identified gaps can inform the policies, systems, and environments to support multi-dimensional literacy skills. These gaps can also inform future literacy research to improve diet quality and health for Americans, particularly for SNAP adults.

The MDFNL model [49] illustrates many factors (e.g., household assets and financial history, geographic location, access to transportation) that may influence the development of different types of food and nutrition literacy. However, our analysis found limited research to understand the magnitude of these effects and potential interacting factors. For example, while it is recognized that low-income populations have lower access to broadband and lower nutrition literacy levels, none of the studies described participants’ access to internet broadband services. There was insufficient information from the 18 studies reviewed to draw conclusions around the impact of geographic location on literacy level.

In general, the 18 studies suggest that adults who have higher food or nutrition literacy scores had better cognitive, behavioral, food security, and/or health outcomes; although, there were no consistent findings across the studies. We had hoped to separately report on the subset of studies that included SNAP adults to identify this population’s unique literacy skills and capacities. However, only six studies [56,58,59,62,68,72] reported including SNAP or SNAP-eligible adults as a sub-population within the larger study population. We did not find sufficient evidence to report on these studies separately.

The instruments and tools used by the 18 studies varied, and most of the studies relied on a tool developed to screen for health literacy. Nearly half of the studies used basic numeracy and reading skills applied to a food product as a proxy for nutrition or health literacy. We identified several limitations of the studies that used the full or shortened version of the NVS tool to assess food and nutrition literacy. Notably, assessing reading and numeracy skills alone does not address the breadth of literacy skills and capacities that individuals need to effectively navigate both in-person and online shopping experiences; this includes the ability to communicate with food retailers or bots and the ability to understand why and how products are marketed to customers throughout their shopping experience, among others. We found the NVS tool to be an incomplete measure of food and nutrition literacy. A recent systematic review documented the inconclusive nature of the available evidence on the association between health literacy and diet quality [77] that necessitates using a more accurate literacy model to assess diet quality.

Our search did not identify any comprehensive food and nutrition literacy tools to assess a range of literacy capacities and skills. Our results concur with those from a previous study [78] that appraised 13 instruments across seven countries to operationalize and measure food and nutrition literacy constructs for adult populations. This study concluded that most of the tools assessed nutrition literacy, and recommended the need to develop multi-dimensional and psychometrically sound measures to capture the broader components of food literacy beyond individuals’ capacities to read and understand food product labels [78]. We recommend that more robust literacy assessment instruments be developed and tested. In particular, these assessment instruments should incorporate digital literacy principles given the growing shift to online platforms to shop for foods and meals, acquire nutrition information (e.g., the new MyPlate app), conduct health screenings, and purchase other diet- and health-related products and resources.

Our study findings coupled with the rapid growth in digital platform use for SNAP and SNAP-Ed participants justify the need for additional research, policies, and actions to support U.S. adults, especially SNAP adults, to make online grocery purchases. This is particularly important during the post-COVID-19 recovery when many SNAP adults are at increased risk for infection and are disproportionately affected by obesity, poverty, food insecurity, and diet-related chronic diseases [39,41,79]. Many institutional actors influence policies and programs that could strengthen the multi-dimensional literacy skills of SNAP. These stakeholders include the U.S. Congress and federal government agencies, such as USDA, HHS, the FCC, the Food and Drug Administration (FDA), the Federal Trade Commission (FTC), and the U.S. Department of Education; digital technology and media firms; and private foundations, academic researchers, professional societies, and civil society organizations. The federal government must re-evaluate the economic livelihoods and digital food and nutrition literacy skills and infrastructure support needed to adequately respond to the pandemic and introduce policies and programs for low-income U.S. populations. Table 4 and Table 5 and the next sections provide specific recommendations for diverse U.S. stakeholders to improve the policies, systems, and environments that support Americans’ digital food and nutrition literacy skills and their digital access, privacy, and safety (Table 4). We also offer recommendations for USDA and other stakeholders to update SNAP and SNAP-Ed policies and programs to improve SNAP adults’ digital food and nutrition literacy skills and digital equity, inclusion and safety (Table 5).

### 4.1. Recommendations for the U.S. Government and Other Actors to Improve the Policies, Systems and Environments That Support Americans’ Digital Food and Nutrition Literacy, and Digital Access, Privacy, and Safety

#### 4.1.1. Digital Food and Nutrition Literacy

This study did not identify any comprehensive literacy tools to measure individuals’ food and nutrition literacy skills. It also did not find any tools that examined individuals’ digital literacy skills related to food retail purchases. Researchers should develop a comprehensive tool to assess Americans’ food and nutrition literacy skills. This effort could be funded by the U.S. government, private foundations, professional societies, or civil society organizations given the importance of better understanding Americans’ multi-dimensional literacy skills and the link between health, digital, food and nutrition literacy, and diet and health outcomes. Private foundations, academic researchers, professional societies, and civil society organizations should also support and conduct external research and evaluations to develop strategies and interventions to improve Americans’ food and nutrition literacy skills. In particular, these interventions could help guide consumers in navigating the digital food retail ecosystem. These actors could also develop policy and practice position statements for members to address digital health, food and nutrition literacy, and digital equity and inclusion for individuals and communities.

HHS defines health literacy in terms of personal and institutional outcomes and has included a research objective in Health People 2030 to increase the health literacy of the U.S. population [80]. However, health literacy and the tools used to test it are not inclusive of food and nutrition literacy. HHS could include food and nutrition literacy skills to promote healthy choices and diets as a priority for future national objectives.

The Centers for Disease Control and Prevention (CDC) provides many resources for educators and childcare providers to support health literacy in children, adolescents, and young adults [81]. However, we did not find any resources to promote food and nutrition literacy in schools despite the recognition that improving health behaviors during childhood and adolescence to prevent chronic disease is easier than doing so in adulthood [82]. The CDC could develop tools and resources for educators to promote food and nutrition literacy in schools to complement the existing health literacy resources. The U.S. Department of Education could also develop national standards for digital food and nutrition literacy for teachers to include in the U.S. school system educational curricula. These national standards could be disseminated and incentivized within community colleges and institutions of higher education to encourage the adoption of curricula to support digital food and nutrition literacy for adults, adapted from existing adult digital learning and literacy resources available through the Literacy Information and Communication System leadership initiative [83].

#### 4.1.2. Digital Access and Safety

We did not identify any studies that measured digital access as it relates to food and nutrition literacy. However, access to the internet and to digital technology are important precursors to individuals’ abilities to develop digital technology skills. The U.S. government has prioritized the expansion of high-speed internet and broadband access for all Americans through the 2021 Build Back Better initiative and a bipartisan infrastructure package. The FCC is also working to subsidize and expand broadband accessibility and affordability for low-income and rural households through the Emergency Broadband Benefit Program and Rural Digital Opportunity Fund [84,85]. These initiatives are addressing the digital divide by strengthening digital equity and inclusion for low-income Americans [86], many of which are SNAP participants. These actions are an important step toward ensuring that all Americans have access to broadband to function effectively in the expanding digital food retail ecosystem.

The growth of digital platform use for food shopping highlights a need to provide education, skills, and legislation to support Americans’ safety, privacy, and security when navigating the digital retail ecosystem. As the Center for Digital Democracy outlined in a 2020 report, online food retailers are able to access and track consumers’ purchase behaviors, location, and personal information and share this information with food manufacturers, advertisers or other third parties, often without consumers’ knowledge [87,88]. This information enables retailers to personalize and target advertisements—the majority of which promote unhealthy food and beverage products—to customers [19,20]. The FTC could examine the marketing practices of online grocery retailers and develop regulatory guidance for the use of automated artificial intelligence (AI) and for the machine learning used to collect this information. The FTC could also update its regulatory guidance for online endorsements, commercial sponsorships, misleading or deceptive advertising and nutrition misinformation shared through social media platforms [89]. This guidance is especially important to protect SNAP adults from the predatory marketing practices of some online retailers, manufacturers and digital technology and media firms [88].

Olzenak et al., 2020 [90] found that online grocers may include some nutrition-related features, such as the ability to filter food-related information by a nutrition attribute. However, the Nutrition Facts panel and ingredient statements were not universally provided for food products on grocery store websites, which the FDA requires for product packaging in brick-and-mortar stores [90]. The FDA should provide regulatory guidance and oversight to ensure that retailers and manufacturers provide clear and understandable Nutrition Facts labels and related product information to enable consumers to make informed purchases online [91].

### 4.2. Recommendations for USDA and Other Stakeholders

#### 4.2.1. Digital Food and Nutrition Literacy

As noted in a 2021 research report [92], following the rapid expansion of the SNAP Online Purchasing Pilot program in response to the pandemic, there has not been any formal evaluation of the program, nor have data been reported on its uptake and use. While the report found that many SNAP participants are purchasing food online, many researchers have also identified barriers to SNAP uptake [92]. Barriers range from a lack of trust in using digital platforms and limited control over the online shopping process to grocery costs and product quality [26,93]. Additional research is needed to understand the barriers that SNAP participants experience when purchasing groceries online, and how to overcome them to improve SNAP Online Purchasing Pilot program participation. Additional research is also needed to compare how the online versus in-person shopping experience may impact SNAP adults’ diet quality and health over time. The USDA, with support from academic researchers, professional societies, private foundations, and civil society organizations, could support research to better understand SNAP adults’ digital access, digital food and nutrition literacy status, and the influence of online food purchasing on long-term diet and health outcomes. The USDA could evaluate the SNAP Online Purchasing Pilot program to identify barriers and gaps to further improve the program.

While six of the identified studies used literacy tools to assess the skills and capacities of SNAP adults, no literacy tools were developed specifically for low-income SNAP adults. Many SNAP adults face unique needs and challenges, such as a lack of social support or lower educational attainment, that may influence their capacity to respond to the standard numeracy- and reading-focused questions used in existing literacy tools. The USDA should support the development, testing, and validation of a tool to assess the multi-dimensional digital food and nutrition literacy status of SNAP adults. The USDA and partners could use the MDFNL model as a foundation to develop this literacy tool. Academic researchers, professional societies, private foundations, and civil society organizations would be key stakeholders to support this process.

The U.S. Congress could authorize and appropriate adequate funding in the 2023 U.S. Farm Bill legislation to support the inclusion of digital food, nutrition, financial, and health literacy skills into SNAP and SNAP-Ed messaging to support policies, systems, and environmental interventions that promote healthy eating patterns [94]. The USDA could incorporate multi-dimensional literacy skills training into SNAP-Ed and provide participants with resources to support their use of digital technology for food purchasing [95].

#### 4.2.2. Digital Equity, Inclusion and Safety

As the USDA expands its use of digital platforms and technology for SNAP and SNAP-Ed, greater attention is needed to address SNAP adults’ access to broadband and digital technologies and the safety measures in place to support them in navigating the digital retail ecosystem. In addition to supporting research to better understand SNAP adults’ barriers to online grocery shopping, the USDA should also implement the Center for Digital Democracy’s recommendations to protect SNAP adults while navigating the online retail ecosystem [88]. These recommendations include clarifying consumers’ privacy rights. The USDA should encourage SNAP-authorized retailers to update their privacy policy disclosures for consumers with limited digital literacy skills to read and understand where and how their personal information is being used by other parties. The USDA should also urge retailers to make privacy polices available in Spanish and other languages. Many retailers, such as Food Lion and Aldi, may partner with companies such as Instacart to promote e-commerce and grocery delivery [96]. The USDA should encourage these companies to comply with updating personal data and geolocation disclosures and should develop additional partnerships through which SNAP users could receive reduced or free delivery for online food purchases.

### 4.3. Study Strengths and Limitations

A strength of this study was the extensive interdisciplinary literature searched to identify the 18 U.S. food and nutrition literacy studies reviewed, and the development of policy-relevant recommendations for the current U.S. political context. A limitation is that, due to time constraints, only one researcher screened articles for inclusion and extracted data for the 18 included studies, which could have introduced bias in the evidence analysis and synthesis. The MDFNL model [49] should be validated and tested in a SNAP-eligible adult population in the U.S. Given the lack of consistency in the findings of the 18 studies, it was not feasible to draw definitive conclusions from these studies. A second limitation was the inability to draw conclusions from the six studies that included SNAP participants or SNAP-eligible adults. Further research is needed to understand the digital food and nutrition literacy skills of SNAP adults, and to develop tools to guide policies and programs that help the SNAP population function in the digital food retail ecosystem.

## 5. Conclusions

Limited literature exists that assesses the nutrition and/or food literacy status of Americans, particularly SNAP adults, and no literature was identified that evaluates Americans’ digital literacy skills and capacities when functioning in an online food retail environment. Existing studies that examine nutrition and/or food literacy only assessed low levels of literacy and did not take into account higher level digital food and nutrition literacy skills needed to function in a e-commerce world. This study identified a need to develop and test more robust literacy assessment instruments that include digital literacy principles. This study also identified a need for greater research, policies, and actions to address the research gaps for Americans’ digital food and nutrition literacy skills and to help SNAP adults navigate the purchase of healthy products online. With online grocery shopping expected to expand given food retailers’ increasing reliance on digital technology, opportunities exist for U.S. government agencies, academia, foundations, and civil society to support research and policies to strengthen MDFNL skills and infrastructure to support a healthy online food retail ecosystem for Americans, especially for SNAP adults.

## Figures and Tables

**Table 1:** The PICOTS framework used to identify relevant food and nutrition literacy studies.

PICOTS	Inclusion	Exclusion
Population	U.S. adults (ages 18 years and older); and/or	Non-U.S. populations
	SNAP participants or SNAP-eligible adults	Children and adolescents
Intervention/Exposure	Nutrition or food literacy measured with specific tool; or Measurement of a food- or nutrition-relevant behavior, such as reading a nutrition label, for which there was a clear link to nutrition or food literacy.	Studies for which the main goal was to develop, assess or confirm the validity or reliability of a specific nutrition or food literacy assessment tool, unless the target population was SNAP adults.
Comparison	Control populations	No comparative population
Outcomes	One or more cognitive, behavioral, food security, or health status outcomes linked to food and/or nutrition literacy, including: *Cognitive:* awareness, knowledge, attitudes, beliefs, self-efficacy, and/or skills. *Behavioral:* food purchasing, preparation, and/or reading a nutrition or product label. *Food Security*: individual- or household-level food security. *Health:* obesity measured by body mass index, type 2 diabetes, heart disease, and/or cancer.	No outcomes relevant to food and/or nutrition literacy reported.
Time	Sources published from inception to 18 January 2021	Sources published after 18 January 2021
Setting/Study Design	Observational, cross-sectional, or intervention studies	Review articles Commentaries or editorials Poster presentations or abstracts Academic theses or dissertations

United States (U.S.); Supplemental Nutrition Assistance Program (SNAP).

**Table 2:** Studies that evaluated the food and/or nutrition literacy of U.S. adults, including SNAP adults, 2006–2021.

Lead Author, Year	Study Objective	Study Location	Study Population	Race/Ethnicity of Study Population	Study Design	Outcomes Measured Cognitive (C); Behavioral (B); Food Security (FS); Health Status (H)	Major Finding
Amuta-Jimenez et al., 2018 [55]	Assess differences between food label literacy and other factors between respondents.	National	U.S. adults (*n* = 3185) with cancer sub-population (*n* = 459); (*F = 280*; *M = 168*)	African American (*n* = 36); Asian (*n* = 7); Caucasian (*n* = 285); Hispanic (*n* = 37); Other (*n* = 9)	Cross-sectional study	C: Food label literacy; confidence in ability to take care of self. B: Food label use; menu use; dietary intake; health information seeking behavior; participation in cancer support group. FS: Not measured. H: Body mass index (BMI).	Food label use associated with better quality diets.
Chang et al., 2017 [56]	Evaluate relationship between nutrition literacy and food insecurity in SNAP participants.	National (house-hold visits with telephone follow up)	U.S. households (*n* = 4158) with SNAP household sub-population (*n* = 1342 weighted)	Black (*n* = 349); Hispanic (*n* = 318); White (*n* = 859); Other (*n* = 148)	Cross-sectional study	C: Knowledge of U.S. nutrition guidelines. B: Food label use; nutrition guideline use; conscientious or frugal buying; financial management practices. FS: Household-level food security. H: Not measured.	SNAP participants face unique financial challenges.
Coffman et al., 2012 [57]	Test the Spanish Nutrition Literacy Scale and assess relationship between literacy and overweight/obesity.	Southeast US city	Spanish-speaking U.S. adults (*n* = 131); (*F = 103*; *M = 28*)	Hispanic (*n* = 131)	Cross-sectional study	C: Nutrition literacy; health literacy. B: Not measured. FS: Not measured. H: BMI (overweight and obesity).	Nutrition literacy scores were lower in overweight or obese respondents.
Gibbs et al., 2016 [58]	Test the Nutrition Literacy Assessment for Parents tool and relationships among parental nutrition literacy, parent, and child BMI and child diet quality.	Kansas City Metropolitan Area	English-speaking U.S. adults in parent-child dyads (*n* = 101); (*F = 86*; *M = 15*) SNAP households (*n* = 25)	Black (*n* = 24); Hispanic (*n* = 6); White (*n* = 70); American Indian or Alaskan Native (*n* = 1)	Cross-sectional study	C: Nutrition literacy by health literacy and nutrition knowledge. B: Dietary intake and quality (parent and child). FS: Not measured. H: BMI (parent and child).	Parental nutrition literacy is a significant predictor of child diet quality.
Grutzmacher et al., 2020 [59]	Examine numeracy skills and Nutrition Fact Label skills in classifying health literacy and NVS performance.	Maryland	SNAP-eligible adults (*n* = 144); (*F = 110*; *M = 34*)	Racial and ethnic data not reported.	Cross-sectional study	C: Health literacy; nutrition literacy. B: Not measured. FS: Not measured. H: Not measured.	The NVS literacy tool appears to measure many skills and constructs simultaneously.
Jay et al., 2009 [60]	Test multimedia intervention to improve food label comprehension in low-income patients with chronic health conditions.	New York City	U.S. English-speaking adult patients with chronic health conditions (*n* = 42); (*F = 34*; *M = 8*)	Asian (*n* = 5); African American (*n* = 11); Caucasian (*n* = 6); Hispanic (*n* = 16); Other (*n* = 2)	Random-ized Interven-tion trial	C: Food label exposure; confidence interpreting knowledge; health literacy. B: Not measured. FS: Not measured. H: Health status measured at baseline but not at post-test.	A multimedia intervention can improve short-term food label comprehension in patients with adequate health literacy.
Jones and Adkins 2021 [61]	Examine associations between nutrition literacy and food selections in school-based food pantry.	Indiana	U.S. adult users of a school food pantry (*n* = 61); (*F = 41*; *M = 20*)	African American (*n* = 12); Caucasian (*n* = 31); Hispanic (*n* = 2); Other (*n* = 16)	Cross-sectional study	C: Nutrition literacy and preferences. B: Food selection at a food pantry. FS: Household-level and child food security. H: Not measured.	Higher adult nutrition literacy was associated with a selection of a more diverse set of food items in a school-based food pantry.
Moore et al., 2020 [62]	Assess and compare nutrition literacy and food insecurity in college students.	Texas (*3 college campuses*)	U.S. adult college students (*n* = 672); (*F = 527*; *M = 69*) **SNAP enrolled (*n* = 14)	Asian (*n* = 96); Black (*n* = 90); Hispanic (*n* = 111); White (*n* = 324)	Cross-sectional study	C: Nutrition literacy. B: Not measured. FS: Individual-level food security. H: Not measured.	Among students with adequate nutrition literacy, a greater proportion were food secure.
Parekh et al., 2018 [63]	Feasibility of nutrition education workshops for cancer survivors to inform the design of a multi-center intervention.	New York City	U.S. adult female English-speaking breast cancer patients post-treatment (*n* = 59)	Asian (*n* = 3); American Indian/Alaskan Native (*n* = 2); Black (*n* = 13); White (*n* = 40); Other (*n* = 1)	Random-ized Interven-tion Trial	C: Nutrition literacy; health literacy. B: Fruit and vegetable, alcohol, and high fiber food intake. FS: Not measured. H: Height; weight.	The workshop interventions were found to be promising and scalable.
Persoskie et al., 2017 [64]	Assess Nutrition Facts label understanding and associations by diet and demographics.	National	U.S. adults (*n* = 3815) Nationally representative (*F = 1893*; *M = 1190*)	Black (*n* = 335); Hispanic (*n* = 478); White (*n* = 2140); Other (*n* = 232)	Cross-sectional study	C: Health literacy. B: Sugar sweetened beverages, fruit, and vegetable intake. FS: Not measured. H: Not measured.	Even with revised or simplified Nutrition Facts label, ability to make calculations was a barrier to greater health literacy.
Rhea et al., 2020 [65]	Test multi- factorial nutrition education skill building program for veterinary medical students to improve food literacy scores.	Louisiana	U.S. adult college veterinary and non-veterinary students (*n* = 37); (*F = 31*; *M = 6*)	Asian (*n* = 6); Black (*n* = 5); Hispanic (*n* = 1); White (*n* = 25)	Four-week interven-tion trial	C: Knowledge and awareness of nutritious foods; food preferences. B: Reads nutrition information before purchase, food selection, food preparation, menu planning, practice cooking skills. FS: Not measured. H: Not measured.	The intervention raised student awareness and increased behaviors to select, prepare, and eat healthy food.
Rosenbaum et al., 2018 [66]	Identify correlates of nutrition literacy; whether nutrition literacy predicted weight loss, food record completion and quality, and session attendance; and associations of race and education.	Mid-Atlantic Metropo-litan Area	U.S. adults with overweight or obesity (*n* = 320); (*F = 250*; *M = 70*)	Black (*n* = 80); White (*n* = 224); Other (*n* = 16)	Secondary data analysis of a six-month behavioral weight loss interven-tion	C: Nutrition literacy. B: Food record completion, food record quality, meeting attendance, self-monitoring. FS: Not measured. H: Weight loss at six months.	Lower nutrition literacy was associated with less weight loss in program participants. Nutrition literacy was lower for Black participants and those with less education.
Roth-man et al., 2006 [67]	Patient comprehension of nutrition labels and relationship of label understanding to patient or demographic characteristics, literacy, and numeracy skills.	TN	U.S. primary health care patient adults (*n* = 200) (*F = 143*; *M = 57*)	Black (*n* = 50); White (*n* = 134); Other (*n* = 16)	Cross-sectional study	C: Nutrition label comprehension, reading, and numeracy skills. B: Food label use and frequency; diet plan use. FS: Not measured. H: Chronic disease status; BMI.	Poor nutrition label comprehension was highly correlated with low literacy and low numeracy skills.
Speirs et al., 2012 [68]	Assess demographic characteristics and the relationship between health literacy and nutrition behaviors.	Maryland	SNAP-eligible adults (*n* = 142); (*F = 108*; *M = 34*)	African American (*n* = 75); White (*n* = 50); Other (*n* = 17)	Cross-sectional survey	C: Health literacy. B: Fruit and vegetable intake; consumption of healthy foods. FS: Not measured. H: Not measured.	Strong relationship between adequate health literacy and healthy consumption behaviors was not found.
Taylor et al., 2019 [69]	Describe the relationship between adherence to distinct dietary patterns and nutrition literacy.	Kansas City Metropolitan Area	U.S. adults (*n* = 386) with diabetes, hypertension, hyperlipid-emia, and/or overweight or obesity; (*F = 274*; *M = 112*)	African American (*n* = 131); White (*n* = 233); Hispanic (*n* = 36); Other (*n* = 46)	Cross-sectional study	C: Nutrition literacy. B: Food intake and practices. FS: Not measured. H: Not measured.	Nutrition literacy predicted diet quality and diet patterns.
Tucker et al., 2019 [70]	Examine results from a culturally sensitive, church-based health promotion intervention among Black adults.	Florida	U.S. adult church congregants (*n* = 321); (*F = 208*; *M = 51*; *no sex reported*; *n = 62*)	Black (*n* = 321)	Random-ized control trial (pre-post interven-tion)	C: Nutrition label literacy. B: Using health promoting behaviors (i.e., healthy eating, healthy drinking, physical activity). FS: Not measured. H: Weight, blood pressure.	Intervention group had significantly greater increases in nutrition label literacy and health behaviors than the control group.
Zoellner et al., 2009 [71]	Examine the nutrition literacy status and preferred nutrition communication channels of adults.	Lower MS Delta Region	U.S. adults (*n* = 177); (*F = 124*; *M = 53*)	African American (*n* = 144); White (*n* = 33)	Cross- sectional study	C: Awareness and exposure to nutrition and health information and communication channels; nutrition literacy. B: Media use for health, food and diet information. FS: Not measured. H: BMI.	Results suggest an association between nutrition-seeking behaviors and nutrition literacy.
Zoellner et al., 2011 [72]	Evaluate health literacy, diet quality, and sugar-sweetened beverage intake while accounting for demographic variables.	Lower MS Delta Region	U.S. adults (*n* = 376); (*F = 287*; *M = 89*) **SNAP-enrolled (*n* = 103)	African American (*n* = 254); non-Hispanic White (*n* = 116); Other (*n* = 6)	Cross-sectional study	C: Health literacy. B: Dietary intake, diet quality (using the Healthy Eating Index), sugary beverage intake. FS: Not measured. H: BMI.	Better understanding of limited health literacy needed to improve practices.

Cognitive (C); Behavioral (B); Food Security (FS); Health Status (HS); United States (U.S.); female (F); male (M); Body mass index (BMI); Mississippi (MS); Supplemental Nutrition Assistance Program (SNAP); Tennessee (TN); U.S. Department of Agriculture (USDA).

**Table 3:** Literacy measurements and tools used in the 18 studies reviewed.

Lead Author, Year	Type of Literacy Measured (Self-Reported)	Literacy Tool Used	Literacy Skills and Capacities Measured	Literacy Proficiency Measured (Based on MDFNL Model)
Amuta-Jimenez et al., 2018 [55]	Food label literacy	Modified Newest Vital Sign (NVS) Health Literacy Screener via Health Information National Trends Survey (mailed)	Ability to use food label to calculate calories, specific nutrients, fat intake, and percent daily value; use of calorie labels on menu.	Functional, Interactive
Chang et al., 2017 [56]	Nutrition literacy	USDA’s National Household Food Acquisition and Purchase Survey	Knowledge of U.S. nutrition guidelines (MyPlate and MyPyramid); try to follow nutrition guideline recommendations; use of nutrition facts panel on food products.	Functional, Interactive
Coffman et al., 2012 [57]	Nutrition literacy; health literacy	Spanish Nutrition Literacy Scale; Short Test of Functional Health Literacy in Adults; NVS Health Literacy Screener	Ability to correctly fill in blanks for food and diet recommendations and health implication statements; ability to read food label and ingredients; ability to use food label to calculate calories, specific nutrients, fat intake, and percent daily value.	Functional, Interactive
Gibbs et al., 2016 [58]	Nutrition literacy	Modified Nutrition Literacy Assessment Instrument	Ability to categorize foods based on dietary recommendations; ability to group foods; knowledge of macronutrients; ability to estimate portion size; ability to read food label and make calculations.	Functional, Interactive
Grutzmacher et al., 2020 [59]	Health literacy	Original and modified NVS Health Literacy Screener	Ability to read food label and ingredients; ability to use food label to calculate calories, specific nutrients, fat intake, and percent daily value.	Functional, Interactive
Jay et al., 2009 [60]	Food label use and understand-ing	Short Test of Functional Health Literacy in Adults (pre/post intervention)	Ability to interpret serving size, fat and nutrient levels, and percent daily values from food labels and compare across labels; confidence in nutrition knowledge.	Functional, Interactive
Jones and Adkins 2021 [61]	Nutrition literacy	Nutrition Literacy Assessment Instrument	Ability to categorize foods based on dietary recommendations; ability to group foods; knowledge of macronutrients; ability to estimate portion size; ability to read food label and make calculations.	Functional, Interactive
Moore et al., 2020 [62]	Nutrition literacy	Modified Nutrition Literacy Assessment Instrument	Ability to answer nutrition questions about energy sources (proteins, carbohydrates, and fats).	Functional, Interactive
Parekh et al., 2018 [63]	Nutrition literacy; health literacy	NVS Health Literacy Screener; Nutrition Literacy Assessment Instrument for Breast Cancer	Ability to categorize foods based on dietary recommendations; ability to group foods; knowledge of macronutrients; ability to estimate portion size; ability to read food label and ingredients; ability to use food label to calculate calories, specific nutrients, fat intake, and percent daily value.	Functional, Interactive
Persoskie et al., 2017 [64]	Health literacy	NVS Health Literacy Screener, shortened version (mailed)	Ability to read food label and ingredients; ability to use food label to calculate calories, specific nutrients, fat intake, and percent daily value.	Functional, Interactive
Rhea et al., 2020 [65]	Food literacy	Eating and Food Literacy Behaviors Questionnaire (pre/post program)	Self-reported purchase and consumption of healthy foods and balanced meals; meal preparation and planning behaviors; use of nutrition information before purchase; use of recipes when preparing meals; food preferences (convenience, taste).	Functional, Interactive
Rosenbaum et al., 2018 [66]	Nutrition literacy	NVS Health Literacy Screener at baseline	Ability to read food label and ingredients; ability to use food label to calculate calories, specific nutrients, fat intake, and percent daily value.	Functional, Interactive
Rothman et al. 2006 [67]	Health literacy	Nutrition Label Survey; Rapid Estimate of Adult Literacy in Medicine; Wide Range Achievement Test, third ed.	Ability to read food label; ability to use food label to calculate various quantities and values, such as nutrient content; ability to compare nutrient contents between two food items.	Functional, Interactive
Speirs et al., 2012 [68]	Health literacy	NVS Health Literacy Screener	Ability to read food label and ingredients; ability to use food label to calculate calories, specific nutrients, fat intake, and percent daily value.	Functional, Interactive
Taylor et al., 2019 [69]	Nutrition literacy	Nutrition Literacy Assessment Instrument	Ability to categorize foods based on dietary recommendations; ability to group foods; knowledge of macronutrients; ability to estimate portion size; ability to read food label and make calculations.	Functional, Interactive
Tucker et al., 2019 [70]	Nutrition label health literacy	NVS Health Literacy Screener	Ability to read food label and ingredients; ability to use food label to calculate calories, specific nutrients, fat intake, and percent daily value.	Functional, Interactive
Zoellner et al., 2009 [71]	Nutrition literacy	NVS Health Literacy Screener; Health Information National Trends Survey-Adapted	Ability to read food label and ingredients; ability to use food label to calculate calories, specific nutrients, fat intake, and percent daily value; use of communication channels for nutrition, food, or diet information; awareness and self-reported knowledge of national dietary guidelines.	Functional, Interactive
Zoellner et al., 2011 [72]	Health literacy	NVS Health Literacy Screener	Ability to read food label and ingredients; ability to use food label to calculate calories, specific nutrients, fat intake, and percent daily value.	Functional, Interactive

Newest Vital Sign (NVS).

**Table 4:** Recommended policies and actions for U.S. government agencies and other stakeholders to improve the policies, systems, and environments that support Americans’ digital food and nutrition literacy, access and safety.

Stakeholder	Recommended Policies and Actions
Government	Centers for Disease Control and Prevention Develop tools and resources for promoting food and nutrition literacy in schools. U.S. Department of Education Develop national standards and resources for digital food and nutrition literacy for inclusion in U.S. schools’ curricula and for adult learners. Encourage community colleges and institutions of higher education to adopt curricula to support digital food and nutrition literacy for adults.
	U.S. Department of Health and Human Services Add a research objective to Healthy People 2030 to increase digital food and nutrition literacy and proficiency of Americans similar to the health literacy objective.
	Food and Drug Administration Ensure that online retailers and manufacturers offer easy access to clear and readable Nutrition Facts labels, ingredient lists, and nutrition information to enable consumers to make informed food and beverage purchases online.
	Federal Trade Commission Examine the marketing practices of online grocery retailers and third-party partners to develop regulatory guidance for the use of automated AI or machine learning that collects and shares customers’ personal information and purchasing patterns. Update regulatory guidance for influencer endorsements, commercial sponsorships, misleading or deceptive advertising, and nutrition misinformation on social media platforms that may target SNAP recipients.
Other	Private Foundations, Academic Researchers, Professional Societies and Civil Society Organizations Develop a comprehensive tool to measure Americans’ digital food and nutrition literacy skills. Support and conduct external evaluations and research on food and nutrition literacy strategies and interventions. Develop policy and practice position statements for members to address digital health, food and nutrition literacy, and digital equity and inclusion comprehensively for individuals and communities.

**Table 5:** Recommended policies and actions for U.S. Congress, USDA and other stakeholders to update SNAP and SNAP-Ed and improve SNAP adults’ digital food and nutrition literacy skills.

Stakeholder	Recommended Policies and Actions
Government	U.S. Congress Authorize and appropriate adequate funding in the 2023 U.S. Farm Bill legislation to include digital food, nutrition, financial, and health literacy messages and interventions within SNAP and SNAP-Ed.
	U.S. Department of Agriculture Support the development, testing, and validation of a common multi-dimensional digital food and nutrition literacy assessment tool for SNAP adults that includes digital technology skills through USDA’s National Institute of Food and Agriculture and Agriculture and Food and Nutrition Research Initiative funding. Incorporate digital, food, nutrition, financial, and marketing literacy skills training into SNAP-Ed and develop resources to support participants’ use of digital technology. Adopt the Center for Digital Democracy’s recommendations to better protect SNAP adults as they navigate the digital food retail ecosystem. Conduct a formal evaluation of the SNAP Online Purchasing Pilot program.
Other	Private Foundations, Academic Researchers, Professional Societies and Civil Society Organizations Support the development, testing, and validation of a multi-dimensional digital food and nutrition literacy assessment tool for SNAP adults. Food Retailers and Third-Party Affiliate Companies Update personal data and geolocation disclosures to make them easier for SNAP adults to read and comprehend where and how their personal information is being used.
